# Effect of Rocking Movements on Afternoon Sleep

**DOI:** 10.3389/fnins.2019.01446

**Published:** 2020-01-21

**Authors:** Rachel M. van Sluijs, Quincy J. Rondei, Diana Schluep, Lukas Jäger, Robert Riener, Peter Achermann, Elisabeth Wilhelm

**Affiliations:** ^1^Sensory-Motor Systems Lab, Institute of Robotics and Intelligent Systems, Department of Health Science and Technology, Swiss Federal Institute of Technology, Zurich, Switzerland; ^2^Sleep & Health Zurich, University Center of Competence, University of Zürich, Zurich, Switzerland; ^3^Medical Faculty, University of Zürich, Zurich, Switzerland; ^4^The KEY Institute for Brain-Mind Research, Department of Psychiatry, Psychotherapy and Psychosomatics, University Hospital of Psychiatry, Zurich, Switzerland; ^5^Institute of Pharmacology and Toxicology, University of Zürich, Zurich, Switzerland

**Keywords:** vestibular stimulation, polysomnography, declarative memory, movement Intervention, nap sleep

## Abstract

**Study Objectives:**

Gentle rocking movements provided by a moving bed have been proposed as a promising non-pharmacological way to promote sleep. In rodents the sleep promoting effect of rocking movements depended on the peak acceleration (named “stimulation intensity”) perceived by the vestibular system. We set out to verify previous reports on the sleep promoting effect of rocking movements and to investigate the importance of stimulation intensity in this process.

**Methods:**

Side-to-side rocking movements along a pendulum trajectory with different peak accelerations (control: 0 m/s^2^, low intensity: 0.15 m/s^2^, medium intensity: 0.25 m/s^2^, high intensity: 0.35 m/s^2^) were provided for 45 min during an afternoon nap opportunity. Participants were assigned to a low intensity group (*n* = 10) experiencing control, low and medium intensity stimulation or a high intensity group (*n* = 12) experiencing control, medium and high intensity stimulation. Sleep and sleep-related memory performance were assessed using polysomnography and a word-pair memory task, respectively.

**Results:**

Participants transitioned faster into deep sleep under the influence of medium intensity rocking as was evident by a faster buildup of delta power compared to the control condition (*n* = 22). The faster buildup did not affect sleep architecture, since e.g., the proportion of the nap spent in deep sleep or latencies did not change. Previously reported effects like a shorter latency to stage N2 and a higher density of sleep spindles were not observed. Sleep quality during control naps of the low intensity group was worse than in the high intensity group. In the low intensity group, we also observed a significant increase in delta power throughout the nap, as well as a higher density of slow oscillations both under the influence of low and medium intensity vestibular stimulation. No such effects were observed in the high intensity group.

**Conclusion:**

Rocking movements may promote nap sleep in young adults. Due to a difference in sleep quality during control naps between the low and high intensity group no conclusion regarding the influence of stimulation intensity were possible. Thus, optimal stimulation settings in humans need further investigation.

## Introduction

Problems with sleep may severely impact our cognitive functioning (Alhola and Polo-Kantola, 2007) and health (Garbarino et al., 2016; Kecklund and Axelsson, 2016). More than one third of the adult population suffers from insufficient sleep or impaired sleep quality (Liu, 2016; Madrid-Valero et al., 2017), with significant consequences for our economy (Hafner et al., 2017). Current therapies for sleep problems include pharmacological (Wilson and Nutt, 2010) and psychological or behavioral therapies, including relaxation strategies (Edinger et al., 2017; Morin et al., 2017). Current pharmacological therapies are not suited for long-term use due to changes in the dose responsiveness (Vinkers and Olivier, 2012) as well as risk of addiction (Konopka et al., 2016). On the other hand, the success of psychological/behavioral highly depends on compliance (Matthews et al., 2013) which restricts their application to a subset of the population. Vestibular stimulation in the form of gentle rocking movements has been proposed as a promising non-pharmacological alternative.

Vestibular stimulation has been used as a soothing and calming intervention during the treatment of various psychiatric and neurological diseases (Grabherr et al., 2015). Several studies, investigating the relationship between vestibular stimulation and sleep, have suggested that motion can be used to alter and possibly promote sleep. Most of these studies have been performed with infants, where quiet rest observed using video or motion tracking devices is taken as a proxy for sleep (Barnard and Bee, 1983; Korner et al., 1990; Johnston et al., 1997). In adults, simultaneous measures of brain activity, eye movement and muscle tone (polysomnography, PSG) give objective insight into changes in sleep architecture and brain activity in relevant frequency bands. When we fall asleep, we usually move from wake (W) into a transitional state (stage N1), followed by sleep (stage N2) which gradually deepens into deep sleep (stage N3). An improvement of sleep can be defined as one or several of the following changes: an increase in the total time asleep from the period in bed (sleep efficiency), a facilitation of the transition from wake to sleep (shorter sleep latency) or from lighter to deeper sleep stages, an increase in the amount or intensity of deep sleep (slow waves), and/or an increase in sleep spindles.

To our knowledge five studies have investigated the effect of rocking motions applied using a moving bed on nap or nighttime sleep in adults reporting different results (Woodward et al., 1990; Bayer et al., 2011; Shibagaki et al., 2017; Omlin et al., 2018; Perrault et al., 2019). Three out of five studies showed a facilitation of the wake-to-sleep transition. This took the form of a shorter latency to sleep onset (first episode of N2) during the first (Perrault et al., 2019) or second consecutive night (Woodward et al., 1990) of sleep with rocking movement. Further, also a shorter duration of sleep stage N1 (Bayer et al., 2011) has been reported as a faster transition from lighter to deeper sleep. A higher total number of spindles was observed three times (Bayer et al., 2011; Omlin et al., 2018; Perrault et al., 2019), twice associated with a higher spindle density (Bayer et al., 2011; Perrault et al., 2019). An increased duration of deep sleep (N3) has been reported (Perrault et al., 2019), concurrent with an increase in the number of slow oscillations typical for deep sleep (Perrault et al., 2019). One study reported a trend toward more time spent in deep sleep (*p* < 0.1) (Shibagaki et al., 2017). Also changes in sleep, which are not necessarily reflecting a promotion of sleep have been observed, including less time spent in N2 (Woodward et al., 1990), increased number of rapid eye movements (Woodward et al., 1990), increased synchronization of slow oscillations and sleep spindles (Perrault et al., 2019), and more time spent in NREM sleep in a subset of aromatherapy tolerant participants (Shibagaki et al., 2017). Sleep efficiency does not seem to be influenced by rocking movements. It thus seems that rocking movements moderately influence various aspects of sleep, possibly depending on the applied stimulation and the study protocol.

**FIGURE 1:**
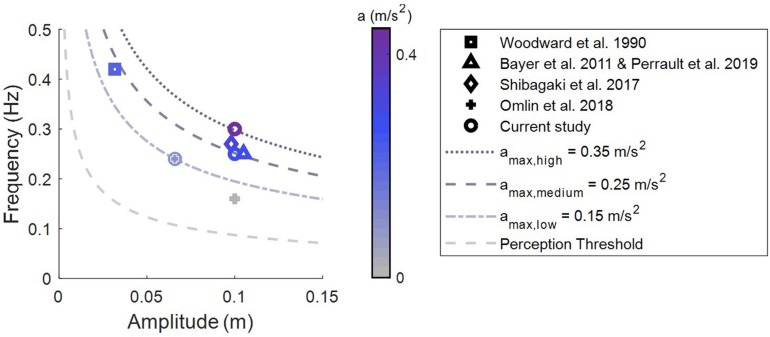
Stimulation intensities (peak acceleration) of rocking movements. Overview of intensities used to assess sleep promoting effects of vestibular stimulation in adults. Lines are isoparametric acceleration curves.

Which vestibular organ is stimulated depends on the movement trajectory shape (linear/rotational/combined) and the movement direction. Beds moved along a pure linear trajectory (Woodward et al., 1990; Shibagaki et al., 2017; Omlin et al., 2018), a parallel swing trajectory (Bayer et al., 2011; Perrault et al., 2019) or a pendulum trajectory (Omlin et al., 2018). Participants were moved either from head-to-toe (Woodward et al., 1990; Shibagaki et al., 2017; Omlin et al., 2018), side-to-side (Bayer et al., 2011; Omlin et al., 2018; Perrault et al., 2019) or up-down (Omlin et al., 2018). Furthermore, the stimulation intensity varied. The intensity of a rocking motion can be quantified by calculating the peak acceleration. As depicted in Figure 1, peak acceleration in previous studies varied from 0.10 to 0.26 m/s^2^.

A study in mice showed that rather than the frequency of the rhythmic stimulation it is the experienced peak acceleration that is crucial for promoting sleep (Kompotis et al., 2019). Stimulation with a peak acceleration of 0.79 m/s^2^ resulted in more NREM sleep at the cost of wake compared to the lower intensity condition (0.32 m/s^2^), while not causing a change in the duration of REM sleep. In their highest intensity condition (1.78 m/s^2^), an increase in NREM sleep was observed at the cost of time spent in both wake and REM sleep, which can no longer be considered a promotion of sleep. Accounting for the sensitivity of the human vestibular system they propose an optimal stimulation range of 0.2–0.26 m/s^2^ (Carriot et al., 2017; Kompotis et al., 2019).

The aim of the current study is to replicate the finding of Bayer et al. (2011) who showed that rocking promotes afternoon sleep in young adults using a larger sample. To this end 45-min naps with and without vestibular stimulation (intensity: 0.25 m/s^2^) were compared. Additionally, we set out to explore the importance of stimulation intensity on the sleep promoting effect of rocking movements in humans. Therefore, all participants had a third nap opportunity with stimulation intensities of either 0.15 m/s^2^ or 0.35 m/s^2^. We hypothesized that rocking movements promote the transition from wake to sleep and increase deep sleep and spindle activity. We expected this sleep promoting effect to be stimulation intensity dependent.

## Materials and Methods

### Sample

Data of twenty-two male participants (Age: 19–31 years, Mean 24.9 years, SD: 3.9 years) were recorded and analyzed. Inclusion criteria were low self-reported susceptibility to motion sickness [adults part of the Motion Sickness Susceptibility Questionnaire (MSSQ) < 19] (Golding, 2006), non-pathological level of daytime sleepiness [Epworth Sleepiness Scale (ESS) < 10] (Johns, 1991; Bloch et al., 1999), no self-reported sleeping or neurological problems, height under 1.90 m and weight under 130 kg (restrictions of the rocking bed). Exclusion criteria were non-compliance (*n* = 1), and not falling asleep during the control nap (*n* = 3). All participants signed informed consent and the Ethical Committee of the Swiss Federal Institute of Technology (EK 2017-N-39) approved the study protocol.

### Vestibular Stimulation

Rocking movements were provided using an automated rocking bed, the Somnomat, which was designed to move smoothly and silently (<30dB, (Crivelli et al., 2014), Figure 2A). For this study the Somnomat was set to generate a sinusoidal oscillation along a pendulum trajectory moving the participant from side-to-side. In this motion the peak acceleration (a_peak_) perceived by the participant depends the frequency of the movement (f, here 0.24–0.3 Hz) and the amplitude of the movement (A; here 0.066–0.1 m). The peak acceleration is determined according to Eq. 1.

**FIGURE 2:**
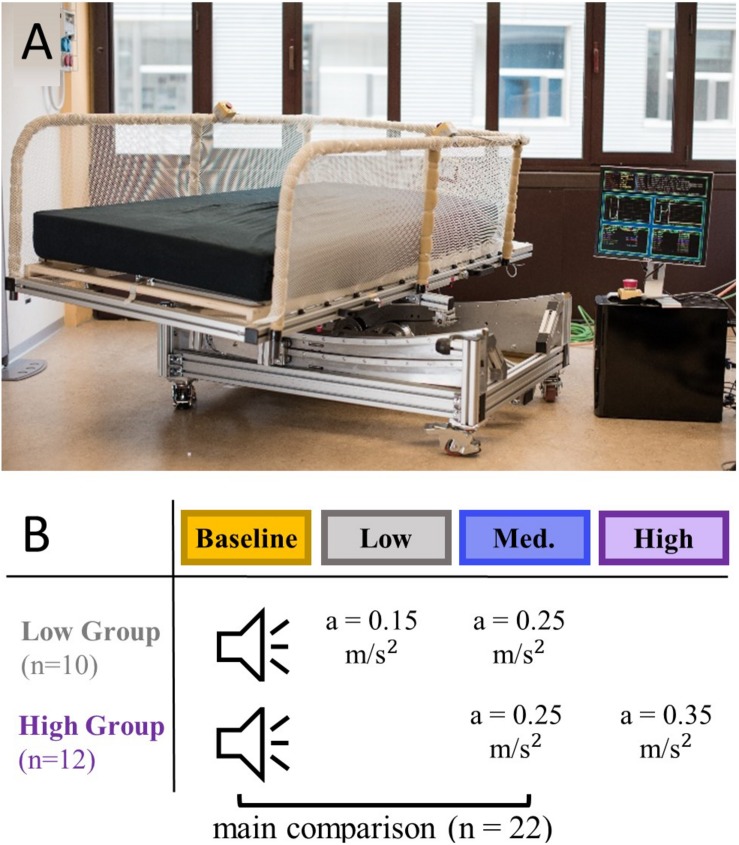
A rocking bed was used to provide vestibular stimulation at three levels of intensity. **(A)** Picture of the Somnomat rocking bed (here not located in the sleep laboratory) performing a side-to-side motion along a pendulum trajectory. **(B)** Participants were divided in a group having a nap opportunity with low intensity stimulation (*n* = 10) and a group having a nap opportunity with high intensity stimulation (*n* = 12), all participants additionally had one control and medium intensity stimulation nap opportunity. Recorded sound of the bed was played back during the control condition.

(1) $$a_{p\,e\,a\,k}=4\,\pi^{2}\,f^{2}\,A$$

### Study Protocol

Participants visited the lab on three occasions for an afternoon nap (light off period 14.15–15.00). Naps were never scheduled on consecutive days and data of the same participant was collected within 1 month. All participants had the opportunity to nap under control conditions (recorded sound of the bed played back) and with medium intensity vestibular stimulation (a_peak_ = 0.25 m/s^2^). In addition, the low intensity group experienced a low intensity vestibular stimulation (a_peak_ = 0.15 m/s^2^) and the high intensity group a high intensity vestibular stimulation (a_peak_ = 0.35 m/s^2^; Figure 2B). The application of the conditions was randomized.

Prior to coming to the lab, participants abstained from alcohol and caffeine consumption for 24 h. Participants were free to choose their own bed and rise times but were instructed to keep these constant the night prior to each measurement. Compliance to regular bedtimes the night prior to all naps was tracked using an actiwatch (Cambridge Neurotechnology Ltd., United Kingdom) worn on the non-dominant wrist. Sleepwatch software (Cambridge Neurotechnology Ltd., United Kingdom) assigned 1-min epochs to mobile or immobile based on integrated activity counts (≥40 corresponds to mobile). Sleep onset (first 10 min period after lights off with consecutive epochs of immobile data, separated by max one epoch of mobility), wake time (last epoch of immobility prior to a 10-min consecutive period of activity around lights on) and the number/duration of wake bouts (series of one or more consecutive mobility epochs) were calculated.

Fifteen minutes before the nap, self-reported sleepiness Stanford Sleepiness Scale (SSS) (Hoddes et al., 1972), anxiety State-Trait Anxiety Questionnaire (STAY) (Knight et al., 1983) and sleep quality the night prior to the nap Groningen Sleep Quality Scale (GSQS) (Meijman et al., 1990) were assessed. A declarative word-pair task, used to assess sleep-related memory performance (Plihal and Born, 1997), was administered 1 h prior to lights off, as well as 30 min after lights on.

During the 45-min lights off period polysomnographic measurements, including electroencephalogram (EEG), electrooculogram and electromyogram of the chin muscles were performed. EEG was recorded according to the 10–20 system (F3, F4, C3, C4, O1, O2, A1, A2) and referenced to Cz (Jasper, 1958). Signals were amplified (Micromed, Mogliano, Veneto, Italy) and filtered with a high pass filter (EEG: −3 dB at 0.15 Hz; EMG: 10 Hz; ECG: 1 Hz) and an anti-aliasing low-pass filter (−3 dB at 67.2 Hz). The signals were sampled and recorded at 256 Hz (Rembrandt DataLab Version 8.0; Embla Systems, Broom field, CO, United States).

### Polysomnography Analysis

The EEG signals were referenced to the contralateral mastoids (A1, A2). Artifacts were marked during visual inspection of the data. Each 20-s epoch was visually attributed to a sleep stage according to AASM criteria by a blinded scorer (Iber et al., 2007).

Latency to stages N1, N2, and N3 were defined as the time from lights off until the occurrence of two consecutive epochs of the respective sleep stage. The duration of initial stage N1 (latency N2 – latency N1) and initial stage N2 (latency N3 – latency N2) were derived from visual scoring. Total sleep period (TSP) is the period from sleep onset (SO; first two epochs of N2) to the last epoch of any sleep stage. Sleep efficiency is the percentage of sleep occurring during the (%TSP) or during the time in bed (%TIB). To assess sleep fragmentation the number of sleep stage changes, as well as the number of excluded epochs (artifacts, movement epochs and epochs with arousals) were derived from the sleep stage scoring.

Electroencephalogram was analyzed in the frequency domain. The signal (C4-A1) was transformed using a Fast Fourier Transform (Hanning window; averages over 5 4-s epochs) and averaged over N2 and N3 epochs (once for all epochs and once only the minimal common number of N2 and N3 epochs across the three conditions within one participant). Furthermore, the temporal evolution of power in the delta frequency range (0.75–4.5 Hz) from 3 min prior to sleep onset to 15 min after sleep onset (SO; latency to N2) was analyzed using a moving mean with a 2-min time window (no separation of sleep stages). Slope of the buildup of delta activity was calculated as delta activity 15 min after SO minus delta activity at SO divided by 15 min.

Individual slow waves with a frequency between 0.4 and 2.4 Hz were detected and characterized (Bersagliere and Achermann, 2010). The signal of the C4-A1 derivation was down sampled to 128 Hz and band-pass filtered in forward and backward direction (third-order Chebyshev type II high-pass filter; –3 dB at 0.4 Hz; sixth-order Chebyshev type II low-pass filter, –3 dB at 2.4 Hz). Positive and negative half-waves were detected when the filtered signal between two consecutive zero-crossings surpassed an amplitude threshold set at 25 μV [corresponding to 37.5 μV in the unfiltered signal (Iber et al., 2007)]. The frequency, duration and amplitude of each half-wave was determined.

Sleep spindles with a frequency between 12 Hz and 15 Hz were detected and characterized (Ferrarelli et al., 2007). The signal of the C4-A1 derivation was down sampled to 128 Hz and band-pass filtered in forward and backward direction (sixth-order Chebyshev type II band-pass filter; –3 dB at 12 Hz; –3 dB at 15 Hz). Individual spindles were detected when the filtered and rectified signal surpassed six times the average amplitude (upper threshold), upon which the start and end time point of the spindle were defined as the first previous and consecutive time point the signal surpassed two times the average amplitude (lower threshold). Only spindles with a duration between 0.5 and 3 s were considered in the analysis (Iber et al., 2007). The average frequency, duration, maximum amplitude, integrated absolute amplitude and activity (integrated absolute amplitude/min) of each spindle were determined. Spindle density was calculated as number of spindles per epoch of NREM sleep.

### Memory Performance Analysis

Participants were instructed to learn the arbitrary association of 40 unrelated word pairs that were presented in randomized order on a computer screen (Plihal and Born, 1997). To test the learned associations one of the two words in each pair was presented again and the participant was instructed to type the second word. Participants were instructed to guess if they were uncertain to ensure an answer was always given. After the participant responded, the correct answer was presented, providing a second learning opportunity. Recall took place directly after learning (immediate recall), as well as 30 min post nap opportunity (delayed recall). One point was given for each correct word pair, and half a point for word pairs with a single/plural error or a typo. The performance improvement from immediate to delayed recall (delayed recall – immediate recall), as well as the initial acquisition rate (immediate recall/delayed recall × 100%), were calculated (Lustenberger et al., 2012).

### Randomization and Statistical Analysis

The order of the conditions was randomized. At the onset of the study only the low intensity protocol was planned, after modification of the protocol to include a high intensity group, participants were assigned randomly to one of the two groups. Since measurements were spread over a 7-months period (21st of August 2018 and 12th of April 2019) and the two experimental groups were not completely randomized in time it was of interest to understand how this might have influenced the results. A correlation between measurement date and sleep time the night prior to the measurements (actigraphy), was observed (pearson correlation, *r* = 0.32, *p* = 0.04). With people sleeping longer closer to the end of the study (winter) than at the beginning of the study (summer). To this end, measurement date was included in the statistical models.

Statistical analysis was performed using RStudio version 1.2.1335 (RStudio Inc., RRID:SCR_000432) using the linear mixed-effects model package lme4 (RRID:SCR_015654). Significance levels for all tests were set to *p* < 0.05. Three participants did not enter stage N3 during one or more naps (low intensity group only) and were therefore excluded from the slow oscillation and spectral analysis that was run over stage N3 sleep.

For the main comparison of sleep during control and medium intensity naps, we used linear mixed-effects models with condition, measurement date and the interaction between measurement date and condition as fixed factors and participant as random factor:

outcome variable∼condition×date+(1∣participant)+ε

To test the significance of the fixed factors an ANOVA was used.

To assess the influence of stimulation intensity we treated the low and high intensity group separately. Within the experimental groups a linear mixed-effects model for interaction between experimental condition and stage on latency to or duration of a specific sleep stage, with participant as random factor, was compared to a null-model without the interaction term, using a χ*^2^* goodness of fit test.

$$\begin{array}{cc} \text{model:}\; & \text{time}∼\text{condition}\times \text{stage}+\left(1|\text{participant}\right)\\ & +\left(1|\text{date}\right)+\varepsilon \\ \text{null-model:}\; & \text{time}∼\text{condition}+\text{stage}+\left(1|\text{participant}\right)\\ & +\left(1|\text{date}\right)+\varepsilon \end{array}$$

*Post hoc* analyses were performed using repeated-measures ANOVA. To compare the two experimental groups prior and during control naps paired *t*-tests were performed.

## Results

### Effect of Rocking Movements (Medium Intensity) on Sleep

To assess whether vestibular stimulation influences sleep we compared control and medium intensity stimulation naps of the full sample (*n* = 22). TIB, TST, sleep efficiency, SO latency, fragmentation index and the number of arousals did not differ between the nights prior to the two naps (Supplementary Table S2). Vestibular stimulation did not influence sleep efficiency during the naps nor the number of sleep stage changes (n.s.) or the number of artifacts (movement artifacts and arousals) (n.s.), suggesting that sleep fragmentation was similar in both conditions.

**FIGURE 3:**
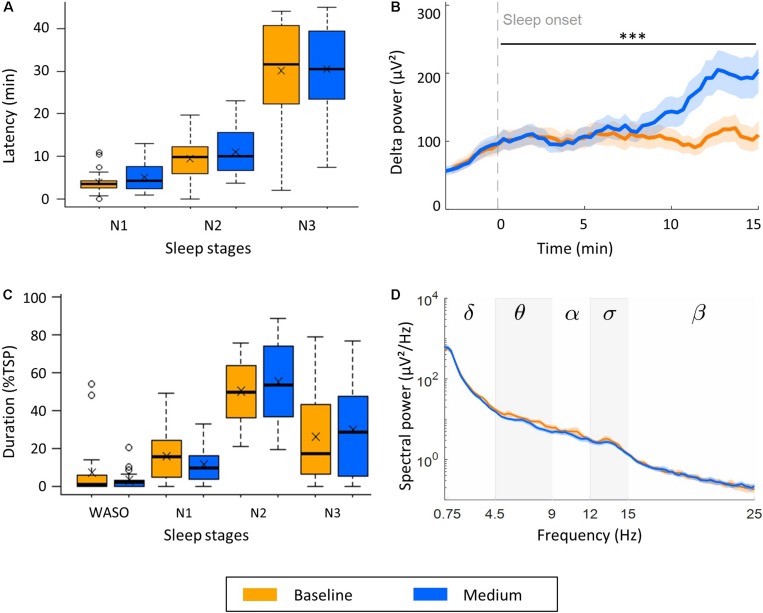
Effect of medium intensity vestibular stimulation on sleep and the sleep EEG (*n* = 22). **(A)** Effect of rocking on transition from wake to NREM sleep stages N1, N2, and N3. **(B)** Buildup of delta power (0.75–4.5 Hz) from 3 min before to 15 min after sleep onset. **(C)** Time spent awake after sleep onset (WASO), and in stage N1, N2, and N3 expressed as percentage of the total sleep period (TSP). **(D)** Power density spectra of channel C4-A1 during stage N3. Boxplots with mean values (*x*) and outliers (o) are illustrated. Lines represent mean power, shading SEM, and stars the significance increase of the slope of delta power (μV^2^/s) during the first 15 min after sleep onset (**B**, ^∗∗∗^*p* < 0.001).

To characterize the influence of rocking movements on the transition from wake to sleep, we assessed the latencies to NREM sleep stages N1, N2, and N3. These latencies were not affected by rocking movements (Figure 3A). Another way of looking at the progressive deepening of sleep is to investigate the buildup of delta power (0.75–4.5 Hz) starting at sleep onset (first occurrence of N2; Figure 3B). The buildup of delta power was significantly faster during medium intensity naps (*M*: 5.95 μV^2^/min, SD: 5.63 μV^2^/min) compared to control naps (*M*: 0.77 μV^2^/min, SD: 3.85 μV^2^/min), *F*(1,40) = 12.45, *p* = 0.001.

The effect of the stimulation on the depth of sleep was investigated by analyzing the period spent in each sleep stage during the TSP, defined as the period from sleep onset to last epoch of NREM sleep. We observed a higher percentage of time spent in consolidated sleep, i.e., stage N2 or N3 during movement naps (*M*: 85.0%TSP, SD: 46.0%TSP) compared to control naps (*M*: 76.4%TSP, SD: 41.5%TSP, Figure 3C), however, this effect was not significant. To assess whether the stimulation influenced the spectral power density of the NREM sleep EEG, we calculated the average EEG power density spectra over the maximal time spend in N3 common in all three naps of each participant (Figure 3D). No significant differences were observed. In line with this observation, the number and density of slow waves (0.4–2.4 Hz) and sleep spindles during stage N3 did not differ between naps (Table 1).

**TABLE 1:** Comparisons between control naps and naps with medium intensity stimulation.

	**Baseline**		**Movement**					
	***M***	**SD**	***M***	**SD**	**df1**	**df2**	***F***	***p***
**Duration (min, *n* = 22)**					**1**		**0.39**	**0.53**
Initial N1	**5.5**	3.4	**6**	3.7	**1**	**20**	**0.42**	**0.52**
Initial N2	**20.6**	9.9	**19.4**	8.5	**1**	**21**	**2.03**	**0.17**
**Sleep fragmentation (*n* = 22)**								
^#^Stage changes	**19.8**	10.2	**17.8**	8.9	**1**	**18**	**1.0552**	**0.3176**
^#^Artifacts	**7.8**	9.1	**9**	9.2	**1**	**19**	**0.6753**	**0.4215**
**Band Power (μV^2^, *n* = 19)**								
δ	**143.9**	75.3	**142.7**	83.3	**1**	**14**	**0.00**	**0.95**
θ	**11.1**	5.5	**9.0**	4.0	**1**	**12**	**0.52**	**0.49**
α	**3.8**	2.2	**3.3**	1.7	**1**	**12**	**0.10**	**0.75**
σ	**3.8**	3.0	**3.2**	2.3	**1**	**14**	**0.16**	**0.69**
β	**0.5**	0.3	**0.5**	0.3	**1**	**14**	**0.01**	**0.93**
**Slow oscillations (*n* = 19)**								
Slow oscillations (#)	**301.3**	355.6	**313.1**	325.5	**1**	**18**	**0.01**	**0.92**
Slow oscillation density (#/20 s)	**9.1**	3.3	**9.1**	3.4	**1**	**14**	**0.13**	**0.72**
Amplitude (μV)	**43.4**	6.8	**44.4**	6.9	**1**	**14**	**0.14**	**0.71**
**Spindles (*n* = 20) and memory task performance (*n* = 18)**								
Spindles (#)	**43.7**	33.2	**48.6**	34.2	**1**	**18**	**0.49**	**0.49**
Spindle density (#/20 s)	**1.7**	0.9	**1.9**	1.0	**1**	**18**	**0.80**	**0.38**
Immediate recall (IR)	**22.4**	6.2	**21.8**	6.3	**1**	**16**	**0.09**	**0.77**
Delayed recall (DR)	**30.0**	6.2	**30.1**	6.3	**1**	**17**	**0.04**	**0.85**
Performance improvement (DR-IR)	**7.6**	3.2	**8.3**	3.4	**1**	**16**	**0.41**	**0.53**
Initial acquisition rate (%) (IR/DR × 100)	**74.2**	11.1	**71.9**	10.8	**1**	**16**	**0.38**	**0.55**

*Sleep stage duration, sleep fragmentation, band power and slow oscillations during N3, and sleep spindles during stage N2 and N3, and memory task performance are reported. A linear mixed model with condition, measurement time and their interaction as fixed factors and participant as random factor was applied. Df1, df2, F and p of the main effect of condition are reported. Bold numbers indicate significant effects of condition (*p* < 0.05). No significant interaction effects were present.*

### Role of Stimulation Intensity

For this part of the analysis the sample was split into a low (*n* = 10) and high (*n* = 12) intensity rocking group. The low and high stimulation intensity groups did not differ with respect to age, body mass index, self-reported general health, subjective sleep quality, daytime sleepiness, susceptibility to motion sickness, or sleep habits (two-sided independent *t*-tests *p* > 0.05, Supplementary Table S1). However, during the control nap sleep quality was lower in the low intensity group than in the high intensity group, since the proportion of the nap spent in deep sleep was lower (*p* < 0.01, Supplementary Table S1) and trends (*p* < 0.1) toward lower sleep efficiency, a longer sleep onset latency and a lower density of sleep spindles were observed. TIB, TST, sleep efficiency, SO latency, and the number of arousals did not differ between the nights prior to the three naps in either group (Supplementary Table S2). However, in the high intensity group the sleep fragmentation index was lower in the night prior to the control nap (*M*: 30.0, SD: 10.8), than prior to the medium intensity (*M*: 35.5, SD: 12.9), and the high intensity nap (*M*: 35.0, SD: 14.4), *p* = 0.04).

Sleep efficiency did not differ between the two groups. However, less stage changes were observed with low intensity (*M*: 13.9, SD: 8.6) and medium intensity (*M*: 16.3, SD: 11.0) rocking compared to the control condition (*M*:23.8, SD: 10.2) in the low intensity group [*F*(2,18) = 6.84, *p* = 0.008]. No such effect was present in the high intensity group (Supplementary Table S2).

**FIGURE 4:**
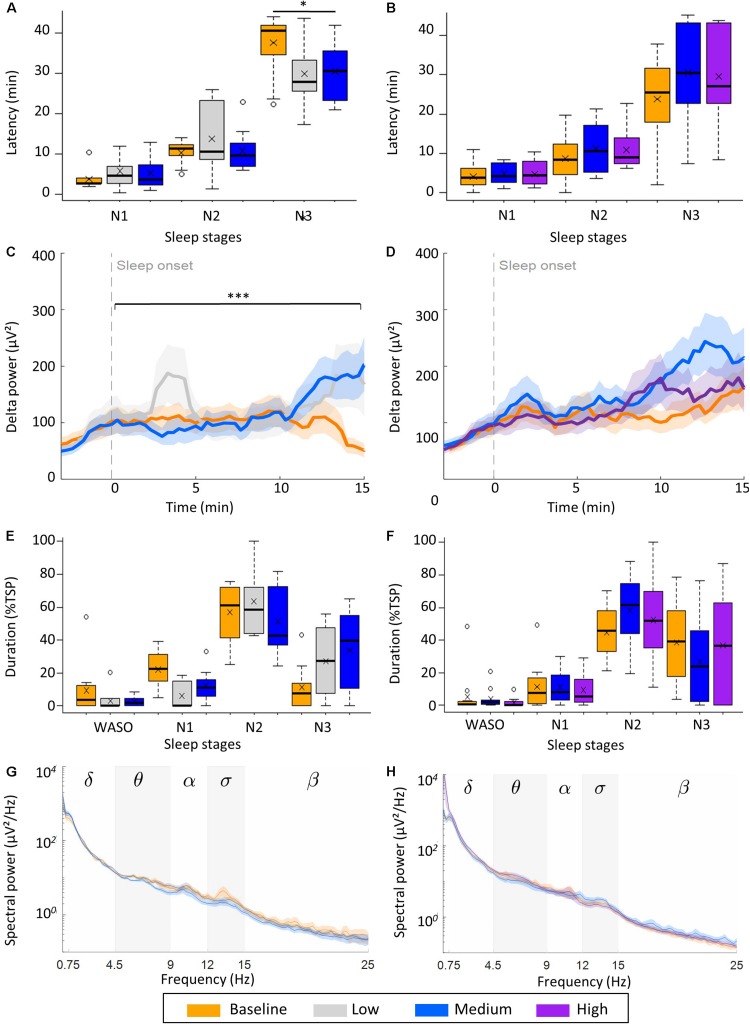
Effect of vestibular stimulation on transition from wake to sleep. **(A,B)** Effect of rocking on transition from wake to NREM sleep stages N1, N2, and N3 (latency). Boxplots with mean value (*x*) and outliers (o) are illustrated shown. **(A)** low intensity (*n* = 10), **(B)** high intensity group (*n* = 12). Boxplots with mean value (*x*) and outliers (o) are illustrated. **(C,D)** Buildup of delta power (0.75–4.5 Hz) from 3 min before to 15 min after sleep onset. **(C)** Low intensity (*n* = 9), **(D)** high intensity group (*n* = 11). **(E,F)** Time spent awake after sleep onset (WASO), and in stage N1, N2, and N3 expressed as percentage of the total sleep period (TSP). Boxplots with mean (*x*) and outliers (°) are shown. **(G,H)** Power density spectra of channel C4-A1 during stage N3. Lines are mean power, shading is SEM of power, stars represent the significance of condition (*p*) of a repeated measures ANOVA of the latency to stage N3 (**A**, ^∗^*p* < 0.01) and of the slope of delta power (μV^2^/min) during the first 15 min after sleep onset (**C**, ^∗∗∗^*p* < 0.001).

While assessing the transition from wake to sleep, we observed a significant interaction between stimulation intensity and sleep stage on the latencies in the low intensity group [χ*^2^*(4) = 20.64, *p* < 0.001; Figure 4A] due to a shorter latency to stage N3 in the low intensity (*M*:30.0 min, SD: 7.5 min), and medium intensity conditions (*M*: 30.5 min, SD: 7.0 min), compared to the control condition (*M*: 37.6 min, SD: 8.2 min, Supplementary Table S3), [*F*(2,18) = 3.98, *p* = 0.038]. The shorter latencies to N3 were due to a shorter duration of initial stage N2 in both the low intensity (*M*: 16.2 min, SD: 3.7 min) and medium intensity condition (*M*: 19.7 min, SD: 7.0 min) than in baseline (*M*: 27.2 min, SD: 9.0 min), [*F*(2,18) = 6.55, *p* = 0.005]. The duration of initial stage N1 was similar in all three conditions. A facilitation of wake to sleep was not observed in the high intensity group, when looking at the sleep latencies or duration of initial stage N1 and N2 (Figure 4B).

In the low intensity group vestibular stimulation significantly accelerated the buildup of delta power during the first 15 min after sleep onset [*F(*2,16) = 13.02, *p* < 0.001, *n* = 9; Figure 4C]. Both during low intensity (*M*: 3.24 μV^2^/min, SD: 5.82 μV^2^/min) and medium intensity stimulation (*M*: 7.08 μV^2^/min, SD: 5.10 μV^2^/min) delta power increased within the 15-min window, while during baseline naps delta power did not increase in the first 10 min and decreased in the last 5 min, resulting in a negative slope (*M*: −3.42 μV^2^/min, SD: 1.38 μV^2^/min). In the high intensity group, a similar buildup of delta power was observed in all three condition, i.e., conditions did not differ (Figure 4D).

In the low intensity group, a significant interaction between experimental condition (control, low intensity or high intensity) and sleep stage on the proportion of time spent in each sleep stage was observed [χ*^2^*(6) = 19.89, *p* = 0.003, Figure 4E]. This was due to a larger proportion of stage N3 sleep [*F*(2,18) = 4.216, *p* = 0.030] at the cost of stage N1 [*F*(2,27) = 6.561, *p* = 0.005] during the two naps with rocking compared to control naps (Figure 4E). Such an effect was not observed in the high intensity group (Figure 4F).

When calculating average power in different frequency bands over all epochs of N3, a significant increase in delta power was observed in the low intensity group [*F*(2,11) = 5.016, *p* = 0.028; Supplementary Table S4]. No effect was present in the high intensity group (Supplementary Table S4). Since delta activity gradually builds up with time, the higher spectral power in the delta band in the low (*M*: 126.1 μV^2^, SD:35.6 μV^2^) and medium intensity (*M*: 118.7 μV^2^, SD: 35.5 μV^2^) condition compared to the control condition (*M*: 106.4 μV^2^, SD: 32.7 μV^2^) might result from later epochs of N3 having more or larger amplitude slow waves. In line with this, a higher number of slow oscillations was observed in movement naps compared to control naps in the low intensity group (Supplementary Table S5). The amplitude of the slow waves did not differ, nor did the duration or the average frequency of slow waves.

In the low intensity group, the number of sleep spindles significantly differed between the three naps, with most sleep spindles occurring during medium intensity stimulation (*M*: 47.00, SD: 23.32), than low intensity stimulation (*M*: 33.11, SD: 19.60) and control naps (*M*: 26.67, SD: 15.93). The spindles were of similar duration, amplitude and frequency (Supplementary Table S6). The increased number was due to increased time spent in NREM sleep, as the density of sleep spindles did not differ between the conditions. Sleep spindles were not affected by rocking in the high intensity group. In line with the absence of a change in sleep spindle density, no impact on the memory task was observed (Supplementary Table S6). Delayed recall, performance improvement and initial acquisition rate were similar in both groups under all conditions.

## Discussion

### Sleep Promoting Effects of Vestibular Stimulation

Vestibular stimulation by slow rocking movements (0.25 m/s^2^) improved sleep. We observed a significant facilitation of the transition from wake to deep sleep in the form of an accelerated buildup of delta power under the influence of rocking. In a subset of the sample, namely participants in the low intensity group, the accelerated transition into sleep additionally led to a larger proportion of the naps spent in deep sleep. These participants also showed more slow waves and a higher level of delta activity during naps with vestibular stimulation compared to naps without stimulation. In the high intensity group, naps with and without stimulation were highly comparable, giving no indication of a promotion or a deterioration of sleep.

### Study Limitations

A limitation of the current study is the observed difference in sleep quality during the control naps of the two experimental groups. It might be that the sleep promoting effect observed in the low intensity and not high intensity stimulation group is due to the difference in control naps between the two groups, rather than due to the stimulation itself (Supplementary Table S1 and Figure 4). However, the low intensity group also took longer to fall asleep and had a slightly shorter, less efficient and more fragmented sleep during the nights prior to the control nap (n.s.). Thus, it is unlikely that the difference in control nap sleep between the experimental groups is due to a difference in sleep pressure resulting from suboptimal sleep quality the night before the measurements. Another factor that might contribute the difference in control naps is the measurement date, since the majority of participants of the low intensity group participated earlier in the study than the participants in the high intensity group and the measurement date was positively correlated with sleep time the night prior to control naps. This could point to a seasonal effect. To correct for such a potential influence, measurement date was taken into account in the statistical analysis.

A further limitation is the chosen study design with a division of subjects in a low and high intensity group that does not allow a statistical comparison of all four conditions. We aimed to recruit 12 participants per experimental group, based on the study of Bayer et al. (2011) who reported statistically significant effects of rocking on nap sleep in a sample of 10 young adults. Based on their reported latency to N2 and accompanying values of spread we conducted power and sample size estimations for a *t*-test for difference between two dependent means. Assuming a between group correlation of 0.7 indicated that a sample size of 21 would give 90% power to detect a difference. Thus, with 22 participants undergoing medium intensity rocking our study had sufficient power and had the largest sample size of the studies reporting on rocking movements and sleep.

### Sleep Promoting Effects Observed in Other Studies

A facilitation from wake-to-sleep onset (Woodward et al., 1990; Perrault et al., 2019) or a shortening of the duration of initial light sleep (Bayer et al., 2011) has been observed before. We additionally report a faster transition from wake to deep sleep. It should be noted that a change in the build-up of delta power under the influence of rocking movements was a change that was not expected, since one previous study did not observe such an effect (Omlin et al., 2018) and the other studies do not report on this. The changes in deep sleep we observed are in line with some, but not all previous studies. One study reported more time spent in deep sleep and more slow oscillations (Perrault et al., 2019), another study more delta power (Bayer et al., 2011). Like in the study by Perrault et al. (2019), vestibular stimulation did not alter the amplitude of the observed slow oscillations in our study, implying that there was a change in the number of slow waves, but not in their amplitude. A facilitation of the wake-to-sleep onset (Woodward et al., 1990; Perrault et al., 2019) or a shortening of the duration of initial light sleep (Bayer et al., 2011) could not be replicated. Also, the increase in the number and density of sleep spindles (Perrault et al., 2019) could not be reproduced.

**FIGURE 5:**
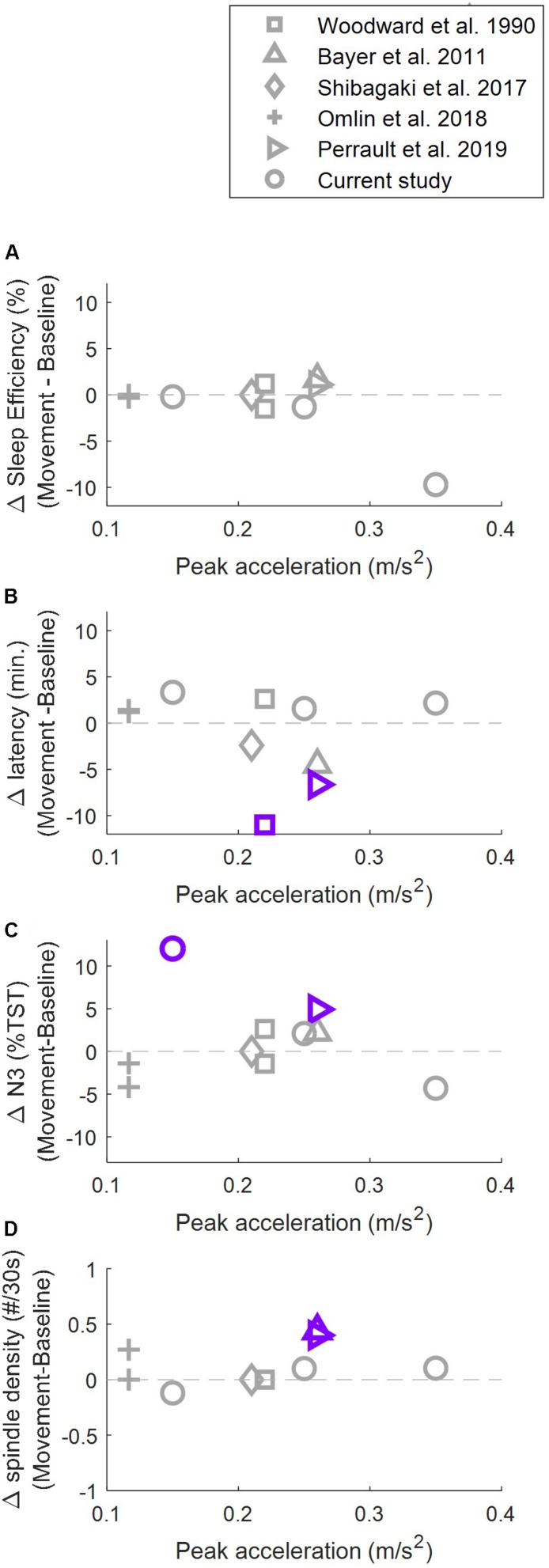
Relationship between stimulation intensity and rocking related changes (rocking – control) in sleep efficiency **(A)**, sleep onset latency **(B)**, proportion of time spent in deep sleep **(C)**, and density of sleep spindles **(D)**. Purple/gray markers indicate significance/no significance at *p* < 0.05. Sleep efficiency was defined as total sleep time/time in bed × 100. Values of(Bayer et al. (2011) were calculated based on the reported total sleep time (TST). Sleep onset latency was defined as time from lights off to the first occurrence of N2, except for Woodward et al. (1990) where sleep onset latency was defined as lights off to first two consecutive minutes of any sleep stage. Bayer et al. (2011) did not report inferential statistics for the latency to stage N2. Proportion of time spent in deep sleep was calculated as% of stage N3 or stages S3+S4 of TST. Density of sleep spindles was defined as spindles per 30 s in NREM sleep, except for Perrault et al. (2019) where the density was calculated only over stage N3. Omlin et al. (2018) calculated the values only during the first 2 h after lights off.)

To better understand the sleep-promoting effect of rhythmic movements and the role which stimulation intensity plays we compared the effect of rocking reported in literature in four main sleep parameters that could indicate a promotion of sleep: sleep efficiency, transition from wake to sleep, proportion of time spent in deep sleep and the density of sleep spindles (Figure 5). No study up to now has observed a significant change in sleep efficiency. For sleep onset latency, proportion of time spent in deep sleep and the density of sleep spindles, significant positive effects of vestibular stimulation have been reported. Importantly, these significant positive effects have been reported under the influence of a wide range of stimulation intensities (from 0.15 to 0.26 m/s^2^). At the same time, several studies where stimulation within this range was provided did not result in a promotion of sleep. This implies that additional factors besides stimulation intensity may play an important role.

Differences between the study protocols of previous studies include the direction of the stimulation (head-to-toe, side-to-side, up-down), the trajectory of the stimulation (linear, parallel swing, pendulum), the timing of sleep (nap vs. nighttime). Furthermore, the rhythmic tactile and auditory experiences (sound of the motor and clicking sounds of the mechanism) differed between rocking beds. The studies controlled for these confounding factors in different ways, with some studies not correcting for this at all, and others playing back the recorded sound or solely keeping the motor running (not correcting for clicking sounds of the mechanism). Sleep might be influenced by rhythmic auditory stimulation (Besedovsky et al., 2017) and likely by some forms of tactile stimulation (Pereira et al., 2017), thus these confounding sensations should be considered with great care while designing studies investigating the influence of vestibular stimulation. Also, Shibagaki et al. (2017) reported a sleep promoting effect in a subset of subjects who responded positively to aromatherapy, an accepted non-pharmacological sleep promoting therapy in Japan, suggesting vestibular stimulation might be a suitable therapy in a subset of the population.

Findings in humans are not in line with findings in rodents. Kompotis et al. (2019) provided vestibular stimulation to mice with a frequency of 1 Hz with three different amplitudes, resulting in three different peak accelerations. They found that low intensity stimulation (mice: 0.32 m/s^2^, human equivalent: 0.8 to 0.11 m/s^2^) did not influence sleep architecture, while medium intensity stimulation (mice: 0.79 m/s^2^, human equivalent: 0.2–0.26 m/s^2^) increased the amount of NREM sleep at the cost of wake. Even more intense stimulation (mice: 1.78 m/s^2^, human equivalent: 0.45–0.59 m/s^2^) led to a further increase in the amount of NREM sleep at the cost of REM sleep, which is no longer considered a promotion of sleep. They suggested that the absence of a sleep promoting effect reported by Omlin et al. (2018) could be explained by the low stimulation intensity. Based on the current results we cannot completely exclude this possibility, since the range of intensities investigated (0.15–0.35 m/s^2^) is not as broad as the one studied in rodents. However, it is important to point out that many negative results were also reported at higher stimulation intensities (Figure 5). Thus, although it is likely that there is a lower limit (sensory perception threshold) and an upper limit (increased risk of motion sickness; unpleasant perception of rocking) of the range of stimulation intensities that promote sleep, there is at this point no evidence for a stimulation intensity dependent effect of vestibular stimulation on sleep in humans.

## Conclusion

Comparing control and movement naps in the full sample we observed a sleep promoting effect of rocking, namely a steeper buildup of sleep intensity after sleep onset. This effect might not depend on the stimulation intensity, as most changes in sleep variables were observed under the influence of both low intensity and medium intensity stimulation. For the low intensity group the medium stimulation (0.25 m/s^2^) led to a smoother transition from wake to deep sleep (shorter duration of initial N2 and shorter latency to deep sleep) and a larger proportion of the sleep period spent in deep sleep, but no significant changes were observed in the high intensity group at the same stimulation. What can be concluded, is that sleep did not deteriorate under the influence of the highest stimulation (0.35 m/s^2^), since sleep efficiency, fragmentation, latency to sleep onset, as well as the amount and intensity of deep sleep were similar to the control nap. Stimulation settings that are best to induce a sleep promoting effect need further investigation.

## Data Availability Statement

The datasets generated for this study are available on request to the corresponding author.

## Ethics Statement

The studies involving human participants were reviewed and approved by the Ethical Committee of the Swiss Federal Institute of Technology (EK 2017-N-39). The patients/participants provided their written informed consent to participate in this study.

## Author Contributions

RS, EW, PA, LJ, and RR designed the study. RS, DS, and QR collected the data. RS and PA performed the data analysis. RS, PA, and EW wrote the manuscript. All authors reviewed the final version of the manuscript.

## Conflict of Interest

The authors declare that the research was conducted in the absence of any commercial or financial relationships that could be construed as a potential conflict of interest.

## References

[B1] Alhola P. Polo-Kantola P. (2007). Sleep deprivation: impact on cognitive performance. *Neuropsychiatr. Dis. Treatm.* 3 553–567. 19300585 PMC2656292

[B2] Barnard K. E. Bee H. L. (1983). The impact of temporally patterned stimulation on the development of preterm infants. *Child Dev.* 54 1156–1167. 10.1111/j.1467-8624.1983.tb00536.x 6354625

[B3] Bayer L. Constantinescu I. Perrig S. Vienne J. Vidal P. P. Mühlethaler M. (2011). Rocking synchronizes brain waves during a short nap. *Curr. Biol.* 21 R461–R462. 21683897 10.1016/j.cub.2011.05.012

[B4] Bersagliere A. Achermann P. (2010). Slow oscillations in human non−rapid eye movement sleep electroencephalogram: effects of increased sleep pressure. *J. Sleep Res.* 19 228–237. 10.1111/j.1365-2869.2009.00775.x 19845847

[B5] Besedovsky L. Ngo H.-V. V. Dimitrov S. Gassenmaier C. Lehmann R. Born J. (2017). Auditory closed-loop stimulation of EEG slow oscillations strengthens sleep and signs of its immune-supportive function. *Nat. Commun.* 8:1984. 10.1038/s41467-017-02170-3 29215045 PMC5719447

[B6] Bloch K. E. Schoch O. D. Zhang J. N. Russi E. W. (1999). German version of the Epworth sleepiness scale. *Respiration* 66 440–447. 10.1159/000029408 10516541

[B7] Carriot J. Jamali M. Chacron M. J. Cullen K. E. (2017). The statistics of the vestibular input experienced during natural self−motion differ between rodents and primates. *J. Physiol.* 595 2751–2766. 10.1113/JP273734 28083981 PMC5390882

[B8] Crivelli F. Heinicke L. Omlin X. Riener R. (2014). “Somnomat: a novel device to investigate the influence of vestibular stimulation on sleep,” in *5th IEEE RAS/EMBS International Conference on Biomedical Robotics and Biomechatronics*, (Piscataway, NJ: IEEE), 774–779.

[B9] Edinger J. D. Leggett M. K. Carney C. E. Manber R. (2017). “Psychological and behavioral treatments for insomnia II: implementation and specific populations,” in *Principles and Practice of Sleep Medicine*, eds Kryger M. H. Roth T. Dement W. C. (Amsterdam: Elsevier), 814–831.

[B10] Ferrarelli F. Huber R. Peterson M. J. Massimini M. Murphy M. Riedner B. A. (2007). Reduced sleep spindle activity in schizophrenia patients. *Am. J. Psychiatry* 164 483–492. 10.1176/ajp.2007.164.3.483 17329474

[B11] Garbarino S. Lanteri P. Durando P. Magnavita N. Sannita W. (2016). Co-morbidity, mortality, quality of life and the healthcare/welfare/social costs of disordered sleep: a rapid review. *Int. J. Environ. Res. Public Health* 13:E831. 10.3390/ijerph13080831 27548196 PMC4997517

[B12] Golding J. F. (2006). Motion sickness susceptibility. *Auton. Neurosci.* 129 67–76. 10.1016/j.autneu.2006.07.019 16931173

[B13] Grabherr L. Macauda G. Lenggenhager B. (2015). The moving history of vestibular stimulation as a therapeutic intervention. *Multisens. Res.* 28 653–687. 10.1163/22134808-00002495 26595961

[B14] Hafner M. Stepanek M. Taylor J. Troxel W. M. Van Stolk C. (2017). Why sleep matters-the economic costs of insufficient sleep: a cross-country comparative analysis. *Rand Health Q.* 6:11. 10.1016/b978-0-12-815373-4.00002-2 28983434 PMC5627640

[B15] Hoddes E. Zarcone V. Dement W. (1972). Development and use of Stanford Sleepiness Scale (SSS). *Psychophysiology* 9:150.

[B16] Iber C. Ancoli-Israel S. Chesson A. Quan S. (2007). *The AASM Manual for the Scoring of Sleep and Associated Events: Rules, Terminology, and Technical Specification.* Westchester, IL: American Academy of Sleep Medicine.

[B17] Jasper H. (1958). Report of the committee on methods of clinical examination in electroencephalography. *Electroencephalogr. Clin. Neurophysiol.* 10 370–375. 10.1016/0013-4694(58)90053-1

[B18] Johns M. W. (1991). A new method for measuring daytime sleepiness: the Epworth sleepiness scale. *Sleep* 14 540–545. 10.1093/sleep/14.6.540 1798888

[B19] Johnston C. C. Stremler R. L. Stevens B. J. Horton L. J. (1997). Effectiveness of oral sucrose and simulated rocking on pain response in preterm neonates. *Pain* 72 193–199. 10.1016/s0304-3959(97)00033-x 9272803

[B20] Kecklund G. Axelsson J. (2016). Health consequences of shift work and insufficient sleep. *BMJ* 355 i5210. 10.1136/bmj.i5210 27803010

[B21] Knight R. G. Waal−Manning H. J. Spears G. F. (1983). Some norms and reliability data for the State−Trait Anxiety Inventory and the Zung Self−Rating Depression scale. *Br. J. Clin. Psychol.* 22 245–249. 10.1111/j.2044-8260.1983.tb00610.x 6640176

[B22] Kompotis K. Hubbard J. Emmenegger Y. Perrault A. Mühlethaler M. Schwartz S. (2019). Rocking promotes sleep in mice through rhythmic stimulation of the vestibular system. *Cur. Biol.* 29 392–401. 10.1016/j.cub.2018.12.007 30686738

[B23] Konopka A. Wysiecka J. P. Samochowiec J. (2016). “Benzodiazepine misuse and addiction. risk factors and adverse behavioral aspects,” in *Neuropathology of Drug Addictions and Substance Misuse*, ed. Preedy V. R. (Amsterdam: Elsevier), 327–333. 10.1016/b978-0-12-800634-4.00032-9

[B24] Korner A. F. Lane N. M. Berry K. L. Rho J. M. Brown B. W. Jr. (1990). Sleep enhanced and irritability reduced in preterm infants: differential efficacy of three types of waterbeds. *J. Dev. Behav. Pediatr.* 11 240–246. 2258442

[B25] Liu Y. (2016). Prevalence of healthy sleep duration among adults-United States, 2014. *Morbidity Mortality Weekly Report* 65 137–141. 10.15585/mmwr.mm6506a1 26890214

[B26] Lustenberger C. Maric A. Dürr R. Achermann P. Huber R. (2012). Triangular relationship between sleep spindle activity, general cognitive ability and the efficiency of declarative learning. *PloS one* 7:e49561. 10.1371/journal.pone.0049561 23185361 PMC3504114

[B27] Madrid-Valero J. J. Martínez-Selva J. M. Couto B. R. D. Sánchez-Romera J. F. Ordoñana J. R. (2017). Age and gender effects on the prevalence of poor sleep quality in the adult population. *Gac. Sani.* 31 18–22. 10.1016/j.gaceta.2016.05.013 27474487

[B28] Matthews E. E. Arnedt J. T. Mccarthy M. S. Cuddihy L. J. Aloia M. S. (2013). Adherence to cognitive behavioral therapy for insomnia: a systematic review. *Sleep Med. Rev.* 17 453–464. 10.1016/j.smrv.2013.01.001 23602124 PMC3720832

[B29] Meijman T. F. Thunnissen M. J. de Vries-Griever A. G. H. (1990). The after-effects of a prolonged period of day-sleep on subjective sleep quality. *Work Stress* 4 65–70. 10.1080/02678379008256965

[B30] Morin C. M. Davidson J. R. Beaulieu-Bonneau S. (2017). “Cognitive behavior therapies for insomnia I: approaches and efficacy,” in *Principles and Practice of Sleep Medicine* Kryger M. Roth T. Dement W. C. (Amsterdam: Elsevier), 804–813. 10.1016/C2012-0-03543-0

[B31] Omlin X. Crivelli F. Näf M. Heinicke L. Skorucak J. Malafeev A. (2018). The effect of a slowly rocking bed on sleep. *Sci. Rep.* 8:2156. 10.1038/s41598-018-19880-3 29391413 PMC5794757

[B32] Pereira S. I. R. Beijamini F. Weber F. D. Vincenzi R. A. Da Silva F. A. C. Louzada F. M. (2017). Tactile stimulation during sleep alters slow oscillation and spindle densities but not motor skill. *Physiol. Behav.* 169 59–68. 10.1016/j.physbeh.2016.11.024 27887994

[B33] Perrault A. A. Khani A. Quairiaux C. Kompotis K. Franken P. Muhlethaler M. (2019). Whole-night continuous rocking entrains spontaneous neural oscillations with benefits for sleep and memory. *Curr. Biol.* 29 402–411. 10.1016/j.cub.2018.12.028 30686735

[B34] Plihal W. Born J. (1997). Effects of early and late nocturnal sleep on declarative and procedural memory. *J. Cogn. Neurosci.* 9 534–547. 10.1162/jocn.1997.9.4.534 23968216

[B35] Shibagaki H. Ashida K. Morita Y. Ikeura R. Yokoyama K. (2017). Verifying the sleep-inducing effect of a mother’s rocking motion in adults. *J. Robot. Netw. Artif. Life* 4 129–133.

[B36] Vinkers C. H. Olivier B. (2012). Mechanisms underlying tolerance after long-term benzodiazepine use: a future for subtype-selective GABAA receptor modulators? *Adv. Pharmacol. Sci.* 2012:41 6864. 10.1155/2012/416864 PMC3321276 22536226

[B37] Wilson S. Nutt D. (2010). “Pharmacological treatment of nocturnal sleep disturbance,” in *Sleep Disorders in Neurology*, eds Overeem S. Reading P. (Hoboken, NJ: Blackwell Publishing Ltd), 55–66.

[B38] Woodward S. Tauber E. S. Spielmann A. J. Thorpy M. J. (1990). Effects of otolithic vestibular stimulation on sleep. *Sleep* 13 533–537. 10.1093/sleep/13.6.533 2281251

