# Supplementary material for: Effect of Rocking Movements on Afternoon Sleep
Source: Front Neurosci. 2020 Jan 21;13:1446. doi: 10.3389/fnins.2019.01446 (PMC6985453; doi:10.3389/fnins.2019.01446)
Supplement: Supplementary file 1 [file Data_Sheet_1.docx]

**Supplementary material**

[**Effect of Rocking Movements on Afternoon Sleep**](https://www.frontiersin.org/Journal/Abstract.aspx?s=1852&name=Sleep_and_Circadian_Rhythms&ART_DOI=10.3389/fnins.2019.01446)

Rachel M. van Sluijs MSc^1,2^*, Quincy J. Rondei MSc^1^, Diana Schluep BSc^1^, Lukas Jäger PhD^1^, Robert Riener PhD^1,2,3^, Peter Achermann PhD^2,4,5^, Elisabeth Wilhelm PhD^1,2^

^1^Sensory-Motor Systems Lab, Institute of Robotic and Intelligent Systems, Department of Health Science and Technology, Swiss Federal Institute of Technology, Zurich, Switzerland

^2^Sleep & Health Zurich, University Center of Competence, University of Zurich, Zurich, Switzerland

^3^Medical Faculty, University of Zurich, Zurich, Switzerland

^4^The KEY Institute for Brain Mind Research, Department of Psychiatry, Psychotherapy and Psychosomatics, University Hospital of Psychiatry, Zurich, Switzerland

^5^Institute of Pharmacology and Toxicology, University of Zurich, Zurich, Switzerland

**Table S1. Demographics, mental state, sleep habits and sleep prior to the baseline nap of the low and high stimulation intensity group.** Susceptibility to motion sickness was assessed using the adults part of the Motion Sickness Susceptibility Questionnaire (MSSQ)(Golding, 2006), daytime sleepiness was assessed using the Epworth Sleepiness Scale (ESS) (Johns, 1991;Bloch et al., 1999), sleepiness was assessed using the Stanford Sleepiness Scale (SSS) (Hoddes et al., 1972), anxiety was assessed using the State-Trait Anxiety Questionnaire (STAY) (Knight et al., 1983) and sleep quality the night prior to the nap was assessed using the Groningen Sleep Quality Scale (GSQS) (Meijman et al., 1988). ** p < 0.01, two-sided independent t-test significant, ^a^ analysed sample for this analysis was n = 9 (low intensity group) and n = 11 (high intensity group).

|  | **Low intensity group**  **(n = 10)**  Mean (SD) | **High intensity group**  **(n = 12)**  Mean (SD) |
| --- | --- | --- |
| ***Demographics*** |  |  |
| Age (years) | 25.3 (4.5) | 24.8 (2.2) |
| Body mass index (kg/m^2^) | 23.4 (2.3) | 22.3 (1.9) |
| General health  (1 = bad, 5= excellent) | 4.4 (0.7) | 4.6 (0.7) |
| Sleep quality  (1 = bad, 5= excellent) | 4.2 (0.4) | 4.0 (0.9) |
| Day time sleepiness  (ESS: 0 = normal, 24 = severe excessive) | 6.2 (4.0) | 7.6 (2.8) |
| Susceptibility to motion sickness  (MSSQ: 0 = not susceptible, 27 = maximally susceptible) | 1.5 (1.3) | 1.1 (1.4) |
| Habitual bedtime (hh:mm) | 23:32 | 23:15 |
| Habitual wake time (hh:mm) | 7:38 | 7:16 |
| Habitual time in bed (h) | 8.1 (0.9) | 8.0 (0.7) |
|  |  |  |
| ***Night prior to baseline nap*** |  |  |
| Time in bed - subjective (h) | 8.0 (0.8) | 8.0 (0.7) |
| Assumed sleep time – actimetry (h) | 7.44 (1.1) | 8.06 (0.6) |
| Sleep efficiency - actimetry (%) | 77.8 (8.1) | 80.0 (8.8) |
| Sleep latency - actimetry (min) | 25 (20.5) | 14 (19) |
| Fragmentation index - actimetry | 40.0 (14.4) | 30.0 (10.8) |
| Sleep quality  (1 = bad, 5= excellent) | 4.2 (0.8) | 3.8 (1.1) |
| Sleep quality  (GSQS: 0 = excellent, 14 = poor) | 2.1 (3.0) | 2.6 (3.1) |
| Sleepiness  (SSS: 1 = wide awake, 7 = sleep onset soon) | 2.8 (1.2) | 2.5 (0.7) |
| State anxiety  (STAY: 20 = minimum, 80 = maximum) | 31.8 (9.5) | 31.0 (7.0) |
|  |  |  |
| ***Baseline nap (polysomnography)*** |  |  |
| Sleep efficiency (sleep time/time in bed %) | 76.4 (14.9) | 85.1 (13.1) |
| Latency to N2 (min) | 10.4 (2.9) | 8.7 (5.2) |
| Proportion of N3 (% total sleep time) ** | 9.9 (11.2) | 36.0 (21.8) |
| Sleep spindle density (#/30s) ^a^ | 2.3 (1.4) | 2.8 (1.3) |

**Table S2. Sleep on the night prior to the nap.** Measures based on actiwatch worn on the non-dominant wrist. Time in bed (TIB), total sleep time (TST), sleep efficiency, sleep onset latency (SOL), fragmentation index and number of arousals are reported. P-values relate to the main effect of condition in a linear mixed-effects model with fixed effects of condition, measurement date and their interaction, and participant as random effect estimated for each outcome variable separately. Stars refer to a main effect of measurement date.

|  | **Baseline** |  | **Low** |  | **Medium** |  |  |
| --- | --- | --- | --- | --- | --- | --- | --- |
|  | **M** | **SD** | **M** | **SD** | **M** | **SD** | **p** |
| TIB (h) | 8.0 | 0.8 | 8.0 | 0.3 | 8.2 | 0.8 | 0.17 |
| TST (h) | 7.4 | 1.1 | 7.5 | 0.7 | 7.6 | 1.0 | 0.98 |
| Sleep efficiency (%) | 83.4 | 6.0 | 79.6 | 10.0 | 89.1 | 4.4 | 0.44 |
| SOL | 5.4 | 10.1 | 13.7 | 12.4 | 9.8 | 12.2 | 0.49 |
| Fragmentation index | 40.0 | 14.4 | 51.9 | 22.5 | 32.4 | 11.4 | 0.39 |
| Arousals (#) | 33.6 | 7.0 | 36.9 | 10.2 | 26.8 | 8.9 | 0.28 |
|  |  |  |  |  |  |  |  |
|  | **Baseline** |  | **Medium** |  | **High** |  |  |
|  | **M** | **SD** | **M** | **SD** | **M** | **SD** | **p** |
| TIB (h) | 8.4 | 0.7 | 8.4 | 0.8 | 7.9 | 1.1 | 0.32 |
| TST (h) | 8.1 | 0.6 | 8.2 | 0.9 | 7.7 | 1.2 | 0.42 |
| Sleep efficiency (%) | 82.9 | 8.1 | 83.0 | 5.7 | 82.6 | 10.7 | 0.41 |
| SOL | 16.3 | 10.8 | 14.5 | 11.5 | 13.2 | 11.7 | 0.45 |
| **Fragmentation index** | 30.0 | 10.8 | 35.5 | 12.9 | 35.0 | 14.4 | 0.04 |
| Arousals (#) | 33.9 | 11.0 | 36.4 | 7.5 | 29.4 | 12.7 | 0.38 |
|  |  |  |  |  |  |  |  |
|  | **Baseline** |  | **Medium** |  |  |  |  |
|  | **M** | **SD** | **M** | **SD** | **p** |  |  |
| TIB (h) | 8.2 | 0.7 | 8.3 | 0.8 | 0.67 |  |  |
| TST (h) | 7.9 | 0.8 | 8.0 | 0.9 | 0.63 |  |  |
| Sleep efficiency (%) | 83.1 | 7.3 | 85.4 | 5.9 | 0.43 |  |  |
| SOL | 12.7 | 11.5 | 12.7 | 11.5 | 0.98 |  |  |
| Fragmentation index | 33.3 | 12.6 | 34.3 | 11.9 | 0.43* |  |  |
| Arousals (#) | 33.8 | 9.6 | 32.7 | 9.1 | 0.87* |  |  |

**Table S3. Sleep Architecture.** Each 20-s epoch of the recordings was visually attributed to a sleep stage according to AASM criteria (Iber et al., 2007) by a blinded scorer. Latencies, durations and stage changes were derived from the visual scoring. Artefacts were marked during visual inspection of the data. A linear mixed-effects model for interaction between experimental condition and stage on latency to or duration of a specific sleep stage, with participant as random factor was compared to a null-model without the interaction term. P-values in the top row represent relate to the comparison of these two models using a χ2 goodness of fit test. P-values within the tables relate to repeated measures ANOVA comparing means of the different conditions. Only p-values below < 0.05 are reported.

|  | **Baseline** | | **Low** | | | **Medium** | | |  | |  | **Baseline** | | **Medium** | | **High** | |  |
| --- | --- | --- | --- | --- | --- | --- | --- | --- | --- | --- | --- | --- | --- | --- | --- | --- | --- | --- |
|  | **M** | ***SD*** | | **M** | ***SD*** | | **M** | ***SD*** | | **p** |  | **M** | ***SD*** | **M** | ***SD*** | **M** | ***SD*** | p |
| **Latencies (min)** |  |  | |  |  | |  |  | | < 0.001 |  |  |  |  |  |  |  |  |
| N1 | 3.8 | *2.4* | | 5.8 | *4.1* | | 5.3 | *4.0* | |  |  | 4.2 | *3.0* | 4.8 | *2.7* | 4.8 | *2.9* |  |
| N2 | 10.4 | *2.9* | | 13.7 | *8.5* | | 10.8 | *5.2* | |  |  | 8.7 | *5.2* | 11.3 | *6.1* | 10.9 | *5.2* |  |
| N3 | 37.6 | *8.2* | | 30.0 | *7.5* | | 30.5 | *7.0* | | 0.037 |  | 23.8 | *9.6* | 30.5 | *12.0* | 29.6 | *11.5* |  |
|  |  |  | |  |  | |  |  | |  |  |  |  |  |  |  |  |  |
| **Duration (min)** |  |  | |  |  | |  |  | | < 0.001 |  |  |  |  |  |  |  |  |
| initial N1 | 6.6 | *3.1* | | 7.9 | *6.6* | | 5.5 | *2.8* | |  |  | 4.5 | *3.5* | 6.4 | *4.5* | 6.1 | *4.0* |  |
| initial N2 | 27.2 | *9.0* | | 16.2 | *3.7* | | 19.7 | *7.0* | | 0.005 |  | 15.1 | *6.9* | 19.3 | *9.8* | 18.7 | *9.6* |  |
|  |  |  | |  |  | |  |  | |  |  |  |  |  |  |  |  |  |
| **Duration (%TSP)** |  |  | |  |  | |  |  | | 0.003 |  |  |  |  |  |  |  |  |
| WASO | 9.4 | *16.5* | | 3.0 | *6.4* | | 2.7 | *3.1* | |  |  | 5.4 | *13.7* | 3.9 | *6.0* | 1.6 | *2.9* |  |
| N1 | 22.1 | *11.5* | | 6.1 | *8.2* | | 12.2 | *9.9* | | 0.005 |  | 11.3 | *14.0* | 11.1 | *10.4* | 9.4 | *10.5* |  |
| N2 | 56.9 | *17.7* | | 63.7 | *21.9* | | 51.1 | *20.2* | |  |  | 44.7 | *15.9* | 58.3 | *22.0* | 52.3 | *25.4* |  |
| N3 | 11.3 | *13.7* | | 27.3 | *22.6* | | 33.9 | *24.8* | | 0.031 |  | 38.7 | *24.1* | 26.7 | *25.8* | 36.7 | *31.9* |  |
| Sleep efficiency | 90.2 | *16.6* | | 97.0 | *6.4* | | 97.2 | *3.2* | |  |  | 94.6 | *13.7* | 96.1 | *6.0* | 98.4 | *2.9* |  |
|  |  |  | |  |  | |  |  | |  |  |  |  |  |  |  |  |  |
| **Sleep fragmentation** |  |  | |  |  | |  |  | |  |  |  |  |  |  |  |  |  |
| # stage changes | 23.8 | *10.2* | | 13.9 | *8.6* | | 16.3 | *11.0* | | 0.006 |  | 16.2 | *9.0* | 19.1 | *6.7* | 16.6 | *6.3* |  |
| # artefacts | 9.0 | *6.5* | | 5.8 | *7.0* | | 6.9 | *6.5* | |  |  | 6.7 | *11.2* | 10.8 | *11.0* | 9.7 | *13.5* |  |

**Table S4. EEG power in several frequency bands during NREM sleep and during stages N2 and N3 separately.** Delta (δ, 0.75 – 4.5 Hz), theta (θ, 4.5 – 9 Hz), alpha (α, 9 – 12 Hz), sigma (σ, 12 - 15 Hz) and beta (β, 15 – 25 Hz) power are reported. P-values relate to repeated measures ANOVA comparing the means of the different conditions. Only p-values below < 0.05 are reported.

|  | **Baseline** | | **Low** | | **Medium** | |  |  | **Baseline** | | **Medium** | | **High** | |  |
| --- | --- | --- | --- | --- | --- | --- | --- | --- | --- | --- | --- | --- | --- | --- | --- |
|  | **M** | ***SD*** | **M** | ***SD*** | **M** | ***SD*** | ***p*** |  | **M** | ***SD*** | **M** | ***SD*** | **M** | ***SD*** | ***p*** |
| **NREM sleep** | **n = 9** |  |  |  |  |  |  |  | n=11 |  |  |  |  |  |  |
| **δ (µV²)** | 59.1 | *30.6* | 67.6 | *26.4* | 83.7 | *25.1* |  |  | 114.7 | *69.9* | 100.1 | *90.8* | 114.0 | *89.1* |  |
| **θ (µV²)** | 8.4 | *2.4* | 8.1 | *1.5* | 7.7 | *1.5* |  |  | 10.1 | *4.9* | 9.5 | *4.8* | 9.8 | *5.3* |  |
| **α (µV²)** | 3.3 | *1.3* | 3.3 | *0.9* | 3.0 | *1.2* |  |  | 3.8 | *1.7* | 3.9 | *1.7* | 3.4 | *1.7* |  |
| **σ (µV²)** | 2.8 | *1.1* | 2.6 | *0.8* | 2.4 | *0.9* |  |  | 3.9 | *2.3* | 4.0 | *2.2* | 3.3 | *2.2* |  |
| **β (µV²)** | 0.6 | *0.3* | 0.6 | *0.2* | 0.5 | *0.2* |  |  | 0.5 | *0.3* | 0.7 | *0.3* | 0.6 | *0.3* |  |
|  |  |  |  |  |  |  |  |  |  |  |  |  |  |  |  |
| **N2** | n = 9 |  |  |  |  |  |  |  | n=11 |  |  |  |  |  |  |
| **δ (µV²)** | 50.2 | *28.0* | 42.5 | *12.5* | 47.6 | *24.1* |  |  | 56.3 | *28.8* | 57.9 | *40.6* | 45.5 | *22.9* |  |
| **θ (µV²)** | 8.0 | *2.6* | 7.5 | *1.7* | 7.3 | *1.9* |  |  | 9.2 | *4.7* | 9.0 | *4.6* | 8.8 | *4.8* |  |
| **α (µV²)** | 3.2 | *1.2* | 3.2 | *0.8* | 3.0 | *1.1* |  |  | 3.9 | *1.7* | 4.0 | *1.7* | 3.7 | *2.4* |  |
| **σ (µV²)** | 2.8 | *1.0* | 2.8 | *0.9* | 2.6 | *0.8* |  |  | 3.8 | *1.7* | 4.2 | *2.4* | 3.5 | *2.1* |  |
| **β (µV²)** | 0.6 | *0.3* | 0.6 | *0.2* | 0.6 | *0.2* |  |  | 0.6 | *0.3* | 0.7 | *0.3* | 0.6 | *0.3* |  |
|  |  |  |  |  |  |  |  |  |  |  |  |  |  |  |  |
| **N3** | n = 9 |  |  |  |  |  |  |  | n=11 |  |  |  |  |  |  |
| **δ (µV²)** | 106.4 | *32.7* | 126.1 | *35.6* | 118.7 | *35.5* | 0.03 |  | 164.4 | *85.0* | 166.7 | *110.8* | 214.9 | *83.4* |  |
| **θ (µV²)** | 9.3 | *1.9* | 9.7 | *1.5* | 7.9 | *1.4* |  |  | 12.0 | *6.6* | 10.0 | *5.5* | 13.0 | *5.6* |  |
| **α (µV²)** | 3.8 | *2.0* | 3.6 | *1.2* | 2.8 | *1.1* |  |  | 3.8 | *2.4* | 3.8 | *2.1* | 3.4 | *1.9* |  |
| **σ (µV²)** | 3.1 | *1.8* | 2.6 | *1.0* | 2.0 | *0.7* |  |  | 4.0 | *3.5* | 4.1 | *3.0* | 3.5 | *3.2* |  |
| **β (µV²)** | 0.6 | *0.4* | 0.5 | *0.2* | 0.4 | *0.2* |  |  | 0.4 | *0.2* | 0.5 | *0.3* | 0.4 | *0.2* | 0.04 |

**Table S5. Slow waves occurring during all NREM sleep and during stages N2 and N3 separately.** P-values relate to a repeated measures ANOVA comparing means of the different conditions. Only p-values below < 0.05 are reported.

|  | **Baseline** | | **Low** | |  | | | **Medium** | |  | |  | **Baseline** | | **Medium** | | **High** | | |  | |  | |
| --- | --- | --- | --- | --- | --- | --- | --- | --- | --- | --- | --- | --- | --- | --- | --- | --- | --- | --- | --- | --- | --- | --- | --- |
|  | **M** | ***SD*** | | **M** | | ***SD*** | **M** | | ***SD*** | |  |  | **M** | ***SD*** | | **M** | | ***SD*** | **M** | | ***SD*** | |  |
| **NREM** |  |  | |  | |  |  | |  | |  |  |  |  | |  | |  |  | |  | |  |
| Count (#) | 194.3 | *212.9* | | 331.0 | | *288.5* | 428.8 | | *233.3* | |  |  | 572.6 | *389.3* | | 447.9 | | *451.6* | 540.5 | | *406.6* | |  |
| Density (#/20s) | 4.0 | *2.1* | | 5.4 | | *2.7* | 6.6 | | *2.3* | | 0.02 |  | 7.1 | *2.9* | | 5.7 | | *3.6* | 6.5 | | *3.1* | |  |
| Frequency (Hz) | 0.7 | *0.1* | | 0.7 | | *0.0* | 0.7 | | *0.0* | |  |  | 0.7 | *0.0* | | 0.7 | | *0.1* | 0.7 | | *0.0* | |  |
| Duration (s) | 1.5 | *0.2* | | 1.4 | | *0.1* | 1.4 | | *0.1* | |  |  | 1.4 | *0.1* | | 1.4 | | *0.2* | 1.4 | | *0.1* | |  |
| Amplitude (µV) | 46.5 | *13.5* | | 44.2 | | *6.5* | 44.6 | | *8.4* | |  |  | 44.7 | *6.0* | | 42.0 | | *8.6* | 46.7 | | *6.8* | |  |
|  |  |  | |  | |  |  | |  | |  |  |  |  | |  | |  |  | |  | |  |
| **N2** |  |  | |  | |  |  | |  | |  |  |  |  | |  | |  |  | |  | |  |
| Count (#) | 96.3 | *68.6* | | 77.7 | | *57.9* | 77.2 | | *42.0* | |  |  | 105.0 | *61.6* | | 166.3 | | *149.6* | 85.9 | | *70.5* | |  |
| Density (#/20s) | 3.0 | *1.2* | | 2.9 | | *0.7* | 3.0 | | *0.7* | |  |  | 3.2 | *1.0* | | 4.1 | | *2.3* | 3.0 | | *1.0* | |  |
| Frequency (Hz) | 0.7 | *0.1* | | 0.7 | | *0.0* | 0.8 | | *0.1* | |  |  | 0.7 | *0.0* | | 0.7 | | *0.1* | 0.7 | | *0.0* | |  |
| Duration (s) | 1.5 | *0.2* | | 1.4 | | *0.1* | 1.4 | | *0.1* | |  |  | 1.4 | *0.1* | | 1.5 | | *0.2* | 1.4 | | *0.1* | |  |
| Ampltude (µV) | 49.4 | *19.5* | | 40.9 | | *7.3* | 39.3 | | *10.6* | |  |  | 40.3 | *6.0* | | 39.0 | | *5.9* | 38.8 | | *4.0* | |  |
|  |  |  | |  | |  |  | |  | |  |  |  |  | |  | |  |  | |  | |  |
| **N3** |  |  | |  | |  |  | |  | |  |  |  |  | |  | |  |  | |  | |  |
| Count (#) | 98.0 | *171.7* | | 253.3 | | *282.5* | 351.6 | | *269.7* | | 0.03 |  | 467.6 | *386.0* | | 281.6 | | *375.0* | 454.6 | | *397.0* | |  |
| Density (#/20s) | 7.8 | *3.0* | | 9.1 | | *3.2* | 9.1 | | *3.0* | |  |  | 9.8 | *3.4* | | 9.1 | | *3.9* | 10.7 | | *2.2* | |  |
| Frequency (Hz) | 0.7 | *0.0* | | 0.7 | | *0.0* | 0.7 | | *0.0* | |  |  | 0.8 | *0.0* | | 0.7 | | *0.1* | 0.7 | | *0.0* | |  |
| Duration (s) | 1.4 | *0.1* | | 1.4 | | *0.0* | 1.4 | | *0.0* | |  |  | 1.4 | *0.1* | | 1.4 | | *0.1* | 1.4 | | *0.1* | |  |
| Ampltude (µV) | 42.0 | *6.4* | | 45.6 | | *7.9* | 42.8 | | *5.4* | |  |  | 44.2 | *7.2* | | 46.0 | | *8.2* | 49.5 | | *8.1* | |  |

**Table S6. Sleep spindles and memory task performance.** Spindles with a frequency between 12 – 15 Hz and a duration between 0.5 and 3 s were automatically detected. The average frequency, duration, peak amplitude, integrated absolute amplitude and activity (integrated absolute amplitude/min) of each spindle were determined. Performance on a word-pair task testing declarative memory. Immediate recall (IR) took place directly after learning the word pairs 1 hour before lights off. Delayed recall (DR) took place 30 min after lights on. Participants who were not native German speakers were excluded from the word-pair task analysis. P-values relate to repeated measures ANOVA comparing means of the different conditions. Only p-values below < 0.05 are reported.

|  | **Baseline** | | **Low** | | | **Medium** | | |  | |  | **Baseline** | | **Medium** | | | **High** | | |  | |
| --- | --- | --- | --- | --- | --- | --- | --- | --- | --- | --- | --- | --- | --- | --- | --- | --- | --- | --- | --- | --- | --- |
|  | **M** | ***SD*** | | **M** | ***SD*** | | **M** | ***SD*** | | **p** |  | **M** | ***SD*** | | **M** | ***SD*** | | **M** | ***SD*** | | **p** |
| **Spindles** | n=10 |  | |  |  | |  |  | |  |  | n=11 |  | |  |  | |  |  | |  |
| Count (#) | 26.67 | *15.93* | | 33.11 | *19.60* | | 47.00 | *23.32* | | 0.02 |  | 57.55 | *37.66* | | 49.82 | *42.22* | | 47.91 | *30.62* | |  |
| Density (#/20s) | 1.56 | *0.91* | | 1.53 | *0.51* | | 1.78 | *0.93* | |  |  | 1.86 | *0.89* | | 1.95 | *1.15* | | 1.84 | *0.68* | |  |
| Duration (s) | 1.13 | *0.26* | | 1.02 | *0.23* | | 1.03 | *0.20* | |  |  | 1.14 | *0.19* | | 1.09 | *0.12* | | 1.10 | *0.14* | |  |
| Peak amplitude (µV) | 34.32 | *53.82* | | 15.08 | *2.35* | | 14.30 | *2.56* | |  |  | 16.28 | *2.76* | | 16.96 | *2.00* | | 15.80 | *2.70* | |  |
| Mean frequency (Hz) | 13.93 | *0.25* | | 13.99 | *0.18* | | 14.00 | *0.27* | |  |  | 14.00 | *0.27* | | 13.88 | *0.28* | | 13.91 | *0.20* | |  |
| Integrated amplitude (µV) | 1518 | *2081* | | 732 | *275* | | 692 | *231* | |  |  | 872 | *252* | | 855 | *167* | | 833 | *256* | |  |
| Activity (µV/min) | 6.99 | *3.15* | | 5.53 | *0.81* | | 5.20 | *0.92* | |  |  | 5.96 | *1.05* | | 6.14 | *0.91* | | 5.80 | *1.06* | |  |
| Power in 12-15Hz range (µV^2^) | 2.54 | *0.89* | | 2.37 | *0.64* | | 2.11 | *0.73* | |  |  | 2.71 | *0.82* | | 2.85 | *1.05* | | 2.46 | *1.01* | |  |
| Power in 11-16Hz range (µV^2^) | 2.75 | *1.08* | | 2.48 | *0.86* | | 2.20 | *0.81* | |  |  | 2.76 | *0.98* | | 2.92 | *1.13* | | 2.54 | *1.18* | |  |
|  |  |  | |  |  | |  |  | |  |  |  |  | |  |  | |  |  | |  |
| **Memory task** | n=9 |  | |  |  | |  |  | |  |  | n=9 |  | |  |  | |  |  | |  |
| Immediate Recall (IR) | 21.8 | *4.7* | | 19.9 | *4.3* | | 21.5 | *6.6* | |  |  | 23.0 | *7.7* | | 22.1 | *6.5* | | 23.6 | *6.8* | |  |
| Delayed Recall (DR) | 28.9 | *6.3* | | 25.8 | *6.9* | | 29.0 | *7.6* | |  |  | 31.2 | *6.2* | | 31.1 | *4.8* | | 30.3 | *6.6* | |  |
| Performance improvement (DR-IR) | 7.1 | *3.5* | | 5.8 | *5.3* | | 7.5 | *2.9* | |  |  | 8.2 | *2.9* | | 9.1 | *3.8* | | 6.8 | *3.5* | |  |
| Initial Acquisition Rate (%) (IR/DR*100) | 76.2 | *10.4* | | 81.2 | *21.1* | | 73.7 | *8.0* | |  |  | 72.2 | *12.0* | | 70.0 | *13.3* | | 77.2 | *11.0* | |  |

**References**

Bloch, K.E., Schoch, O.D., Zhang, J.N., and Russi, E.W. (1999). German version of the Epworth sleepiness scale. *Respiration* 66**,** 440-447.

Golding, J.F. (2006). Motion sickness susceptibility. *Autonomic Neuroscience* 129**,** 67-76.

Hoddes, E., Zarcone, V., and Dement, W. (1972). Development and use of Stanford Sleepiness Scale (SSS). *Psychophysiology*.

Iber, C., Ancoli-Israel, S., Chesson, A., and Quan, S. (2007). *The AASM manual for the scoring of sleep and associated events: rules, terminology, and technical specification.* Westchester, IL: American Academy of Sleep Medicine.

Johns, M.W. (1991). A new method for measuring daytime sleepiness: the Epworth sleepiness scale. *Sleep* 14**,** 540-545.

Knight, R.G., Waal‐Manning, H.J., and Spears, G.F. (1983). Some norms and reliability data for the State‐Trait Anxiety Inventory and the Zung Self‐Rating Depression scale. *British Journal of Clinical Psychology* 22**,** 245-249.

Meijman, T., De Vries-Griever, A., De Vries, G., and Kampman, R. (1988). The evaluation of the Groningen sleep quality scale. *Groningen: Heymans Bulletin* 2006.
